# The Kunitz-Type Protein ShPI-1 Inhibits Serine Proteases and Voltage-Gated Potassium Channels

**DOI:** 10.3390/toxins8040110

**Published:** 2016-04-13

**Authors:** Rossana García-Fernández, Steve Peigneur, Tirso Pons, Carlos Alvarez, Lidice González, María A. Chávez, Jan Tytgat

**Affiliations:** 1 Centro de Estudio de Proteínas, Facultad de Biología, Universidad de la Habana, Calle 25 No. 455, 10400 La Habana, Cuba; rossana.gf@gmail.com (R.G.-F.); calvarez@fbio.uh.cu (C.A.); libelulalid@gmail.com (L.G.); 2 Laboratory of Toxicology and Pharmacology, KU Leuven, Campus Gasthuisberg O&N2, Herestraat 49, P.O. Box 922, B-3000 Leuven, Belgium; steve.peigneur@pharm.kuleuven.be; 3 Structural Biology and Biocomputing Programme, Spanish National Cancer Research Centre (CNIO), C/Melchor Fernández Almagro 3, Madrid E-28029, Spain; tpons@cnio.es

**Keywords:** protease inhibitor, Kv channel inhibitor, sea anemone, toxin, Kunitz-type protein

## Abstract

The bovine pancreatic trypsin inhibitor (BPTI)-Kunitz-type protein ShPI-1 (UniProt: P31713) is the major protease inhibitor from the sea anemone *Stichodactyla helianthus*. This molecule is used in biotechnology and has biomedical potential related to its anti-parasitic effect. A pseudo wild-type variant, *r*ShPI-1A, with additional residues at the *N*- and *C*-terminal, has a similar three-dimensional structure and comparable trypsin inhibition strength. Further insights into the structure-function relationship of *r*ShPI-1A are required in order to obtain a better understanding of the mechanism of action of this sea anemone peptide. Using enzyme kinetics, we now investigated its activity against other serine proteases. Considering previous reports of bifunctional Kunitz-type proteins from anemones, we also studied the effect of *r*ShPI-1A on voltage-gated potassium (Kv) channels. *r*ShPI-1A binds Kv1.1, Kv1.2, and Kv1.6 channels with IC_50_ values in the nM range. Hence, ShPI-1 is the first member of the sea anemone type 2 potassium channel toxins family with tight-binding potency against several proteases and different Kv1 channels. In depth sequence analysis and structural comparison of ShPI-1 with similar protease inhibitors and Kv channel toxins showed apparent non-sequence conservation for known key residues. However, we detected two subtle patterns of coordinated amino acid substitutions flanking the conserved cysteine residues at the *N*- and *C*-terminal ends.

## 1. Introduction

Cnidaria is an ancient clade of animals whose venom arsenal comprises biologically-active compounds, such as proteases inhibitors and toxins, acting on ion channels, such as the transient receptor potential family member vanilloid 1 (TRPV1) and voltage-gated sodium and potassium channels [1,2,3,4]. Some toxin family types found in sea anemones have been previously identified in other venomous organisms. The most striking examples are peptides from the Kunitz-type family, also found in cone snails, insects, scorpions, spiders, reptiles and ticks. Most studies of Cnidarian BPTI-Kunitz-type peptides are focused on their antiproteolytic effect [5,6]; however, their toxic effect has been poorly investigated compared to similar proteins from other venomous animals.

ShPI-1 (UniProt ID: P31713) is a well-known protease inhibitor isolated from the Caribbean Sea anemone *Stichodactyla helianthus*, containing 55 amino acids cross-linked by three disulfide bridges. This inhibitor has a broad specificity toward several serine proteases, *i.e.*, trypsin, chymotrypsin, human neutrophil elastase, kallikrein and plasmin, and can bind aspartic and cysteine proteases, such as pepsin and papain, respectively [7]. A variant of ShPI-1 (*r*ShPI-1A) containing the *N*-terminal tag EAEA has been obtained to favor expression in the yeast *Pichia pastoris* [8]. This variant also contains two *C*-terminal residues (LG) that remained as part of the cloning procedure. Despite these differences, the X-ray structure of *r*ShPI-1A is highly similar to the NMR structure of the molecule purified from the natural source, thus demonstrating that the additional residues do not affect the native fold [9]. Preliminary functional studies also revealed that these tags do not affect the specificity and strength of trypsin inhibition [8]. Hence, the pseudo wild-type inhibitor *r*ShPI-1A may be used for structure-function relationship studies of ShPI-1, as well as for developing its already reported biotechnological and biomedical applications [8,10,11].

ShPI-1 shares structural homology with both the very potent Kunitz-type protease inhibitor BPTI and snake dendrotoxins (DTXs), which are powerful blockers of voltage-gated potassium channels (K_V_) [12,13]. Five similar proteins from sea anemones have been shown to possess a dual function, since they inhibit serine proteases and K_V_ channels. Kalicludines (AsKC1-AsKC3) from *Anemonia sulcata* are the first bifunctional BPTI-Kunitz-type proteins isolated from sea anemones [14]. Similar activities were later reported for SHTX-III from *Stichodactyla haddoni* and APEKTx1 from *Anthopleura* *elegantissima* [15,16]. In general, little is known about the Kv channel subtype selectivity of these type 2 toxins. A broad screening of different ion channels reported for the first time for APEKTx1 revealed a potent activity against K_V_1.1 with IC_50_ values in the lower nanomolar range and pointed out an impressive selectivity for these channels over the other isoforms tested [16]. Such high molecular specificity and potency has long made venoms a promising source of drug candidates. Moreover, these molecules are valuable tools to study the structure and function of potassium channels and also to be used for drug development.

*S. helianthus*, which lives on Caribbean reefs and uses its soft green tentacles to stun shrimp with a cocktail of toxins, is the natural source of ShK, a type 1 potassium channel toxin that targets K_V_1.1, K_V_1.3, K_V_1.6, K_V_3.2 and K_Ca_3.1 channels [17]. New analogs of this molecule with improved selectivity and pharmaceutical potentialities have been obtained, being perhaps the most compelling venom-derived drug in development [18,19]. However, up to date, only two ion channel toxins, Shk and the type 2 sodium channel toxin Sh1 [20], have been described in *S. helianthus*.

Here, together with a deeper functional characterization of the antiproteolytic activity of *r*ShPI-1A, we demonstrated that this molecule is a very potent voltage-gated potassium channel blocker, able to bind K_V_1.1, K_V_1.2 and K_V_1.6 channels. Of note, ShPI-1 may be a new member of the type 2 potassium channel toxins family from sea anemones. Sequence and structural comparison of ShPI-1 and other Kv channel toxins revealed two subtle patterns of coordinated amino acid substitutions [*X*_*a*−2_*X*_*a*−1_Cys^I^*X*_*a*+1_*X*_*a*+2_*X*_*a*+3_] around the first cysteine residue (Cys^I^) and [Cys^V^*X*_*b*+1_*X*_*b*+2_*X*_*b*+3_Cys^VI^] that contain the Cys^V^ and Cys^VI^ residues toward the *C*-terminal of Kv blocking toxins sharing a BPTI-Kunitz fold. Both patterns contain key residues for inhibition of potassium channels and are close in space to each other, constituting a contact surface.

## 2. Results and Discussion

### 2.1. rShPI-1 Specificity and Dissociation Constants against Serine Proteases

The pseudo wild-type inhibitor *r*ShPI-1A has been previously characterized by our group, but mainly in terms of specificity and *Ki* values against trypsin [8]. Now, we extended the functional characterization of this recombinant molecule by determining its specificity and *Ki* values against other serine proteases previously tested with the natural inhibitor [7], besides factor *Xa* that has not been formerly evaluated.

**Figure 1:**
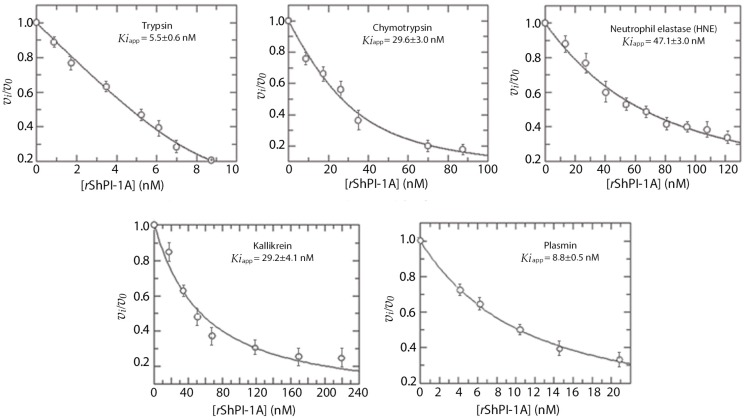
Protease inhibition curves using *r*ShPI-1A. Fixed concentrations of each enzyme were mixed with increasing concentrations of *r*ShPI-1A (displayed in nM). Each enzymatic activity was determined after adding the corresponding substrate (see Materials and Methods for experimental details). The fractional enzymatic activities (*v_i_*/*v*_0_) were calculated after measuring the initial reaction rates with (*v_i_*) and without inhibitor (*v*_0_). Each connecting line represents the best fits to the quadratic Morrison equation for tight binding inhibitors described in [21]. Apparent *Ki* values (*Ki*_app_) were calculated by adjusting the experimental points to that equation and are shown here as the mean ± SE (*n* = 3).

As observed in Figure 1, *r*ShPI-1A affected the enzymatic activities of trypsin-like enzymes, *i.e.*, pancreatic trypsin, plasmin and kallikrein. It also inhibited the activity of chymotrypsin and human neutrophil elastase (HNE). Binding to the latter is in contrast with the absence of activity against pancreatic elastase (Table 1), a behavior also detected in the natural molecule [7]. The concave inhibition curves obtained under experimental conditions of [*E_o_*]/*Ki* = 1–10 indicated that *r*ShPI-1A is a reversible inhibitor of pancreatic trypsin, as previously described [8], as well as of chymotrypsin, HNE, plasmin and kallikrein. This behavior allowed determining the apparent *Ki* values (Figure 1).

Real *Ki* values (Table 1) were then calculated considering the substrate concentration and indicated that *r*ShPI-1A is a tight-binding inhibitor (*Ki* ≤ 10^−7^ M [22,23]) of the five enzymes. Preincubation of *r*ShPI-1A with these serine proteases over varying periods of time did not affect its inhibitory capacity, suggesting that the maximal inhibitory capacity is reached within the first minute of incubation. Moreover, in all cases, the fractional enzymatic activity (*v_i_*/*v*_0_, meaning the ratio between the initial reaction rates with (*v_i_*) and without inhibitor (*v*_0_)) increased when substrate concentration was increased. This demonstrates that the substrates induce enzyme-inhibitor complex dissociation, thus corroborating the reversibility.

The interaction of ShPI-1 with its targeted proteases proceeds through the canonical mechanism involving mainly the reactive P1 site of the primary binding loop [24]. Moreover, the basic residue Lys13 at the P1 site is the main determinant for tight binding inhibition of trypsin-like enzymes [24,25]. The lower inhibition strength against kallikrein, compared to trypsin and plasmin, was expected since the kallikrein S1 pocket accommodates Arg over Lys residues at the P1 site better [26]. This is related to the substitution of Ser190, found in trypsin and plasmin, by Ala. Consequently, the S1 pocket of kallikrein lacks a hydrogen-bond partner for P1 Lys, which does not bind as tightly as Arg at the inhibitor’s P1 site [27]. A similar, but even more marked preference for Arg is found in other trypsin-like proteases from the coagulation pathway, e.g., α-thrombin and factor Xa, since their S1 pockets, also having Ser190, are slightly more hydrophobic and larger. This is consistent with the lack of activity of *r*ShPI-1A against these two enzymes, observed even using a high [*I*_0_]/[*E*_0_] ratio and increasing the incubation times up to 30 min (Table 1).

The inhibition strength against serine proteases with other specificities, *i.e.*, HNE and chymotrypsin, was also lower (*Ki* 10^−8^ M) compared to trypsin. According to X-ray crystallography, the interaction with chymotrypsin (PDB ID: 3T62) proceeds through a different conformation (named up-conformation) of the P1 site at the entrance of the enzyme S1 pocket, as previously reported for similar inhibitors [28]. The mode of accommodation in the catalytic pocket of HNE is still unknown, but molecular modeling studies have suggested that polar desolvation and the presence of Asp226 in HNE could favor the interaction with the basic P1 residue. These interactions cannot be established by Thr226 of PPE, which precludes the insertion of K13 side-chain into the S1 subsite of this enzyme [29], thus explaining the absence of activity against this enzyme.

### 2.2. Activity toward Kv Channels: Electrophysiological Recordings

**Figure 2:**
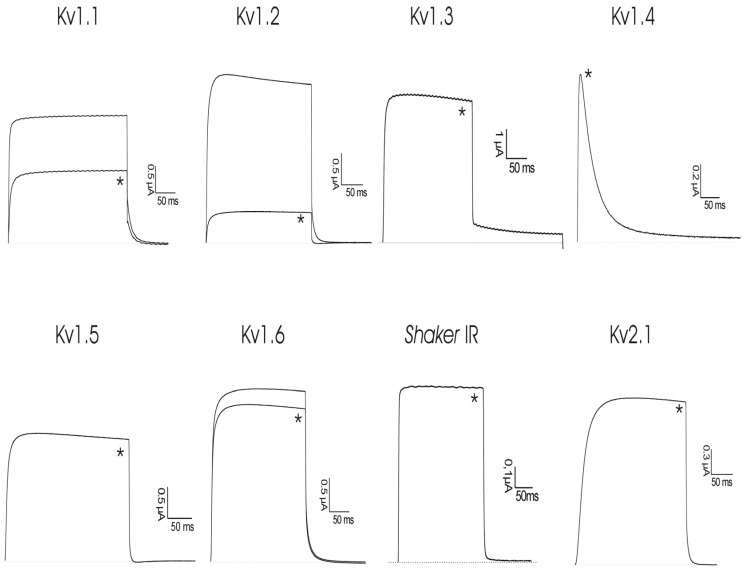
Activity of *r*ShPI-1A on ion channels expressed in *X. laevis* oocytes. Traces are representative of at least three independent experiments (*n* ≥ 3). The dotted line indicates the zero current level. The asterisk (*) distinguishes the steady-state current after application of 50 nM peptide.

*r*ShPI-1A was subjected to a screening on eight cloned voltage-gated potassium channels (rKv1.1, rKv1.2, hKv1.3, rKv1.4, rKv1.5, rKv1.6, Shaker IR, hKv2.1) (Figure 2). Although only lower concentrations of peptide were tested, 50 nM of *r*ShPI-1A was able to block Kv1.1, Kv1.2 and Kv1.6 channels. The same concentration had no effect on other Kv channel isoforms from the *Shaker* (Kv1.3, Kv1.4-Kv1.6 and *Shaker* IR) or *Shab* (Kv2.1) families. Concentration response curves were constructed to determine the values at which half of the channels were blocked by *r*ShPI-1A. The IC_50_ values obtained are shown in Table 2. Kv1.2 channels were used to further investigate the characteristics of inhibition. The inhibition of Kv1.2 channels induced by the toxin was not voltage dependent, as in the range of test potentials from −30 mV to +30 mV, no difference in the degree of blockade could be observed.

**Figure 3:**
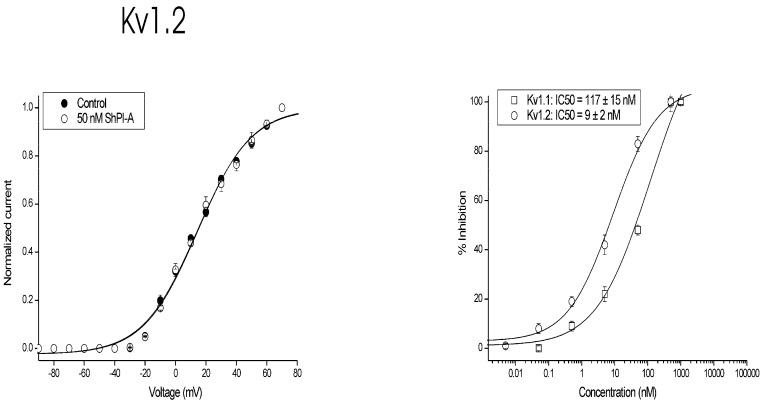
Characterization of *r*ShPI-1A activity on channel gating. **Left panel**: current-voltage relationship. Closed symbols are the control condition; open symbols are after application of 50 nM peptide. **Right panel**: concentration-response curve on Kv1.1 and Kv1.2 channels obtained by plotting the percentage of blocked current as a function of increasing toxin concentrations. All data represent at least three independent experiments (*n* ≥ 3) and are presented as the mean ± standard error.

To investigate whether the observed current inhibition is attributed to obstruction of the pore rather than to altered channel gating upon toxin binding, the IV curves were constructed (Figure 3). The IV curves in the control and in the presence of 50 nM peptide were characterized by V_1/2_ values of 14 ± 2 and 15 ± 2 mV (*n* ≥ 3), respectively. It can be concluded that no significant shift in the midpoint of activation occurred (*p* < 0.05). Altogether, these experiments imply that current inhibition upon *r*ShPI-1A binding does not result from changes in the voltage dependence of channel gating. Upon washout the current recovered quickly and completely (data not shown), suggesting that inhibition of Kv1.2 channels occurred rapidly and that its binding was reversible.

According to these results, *r*ShPI-1A, a recombinant wild-type variant structurally similar to the canonical serine protease inhibitor ShPI-1 from the sea anemone *S. helianthus*, is a potent Kv channel inhibitor. This behavior indicates that ShPI-1 is a new member of the type 2 sea anemone toxins family. Compared to the other five reported members of this family known to target also a serine protease, *i.e.*, trypsin (Table 2), *r*ShPI-1A is unique in its ability to strongly inhibit a broader spectrum of serine proteases, together with a potent activity against more than one Kv channel, *i.e.*, Kv1.1, Kv1.2 and Kv1.6 channels.

The main strategy of venomous animals in either defense or predation situations is prey immobilization through a mix of compounds exhibiting a variety of bioactivities and functions, such as protease inhibitors, epidermal growth factor (EGF)-like peptides (*i.e.*, gigantoxin in *S. gigantea*), phospholipases A2, cytolysins and ion channel modulators. Peptide fingerprint data of a neurotoxic pool from *S. helianthus* revealed a total of 113 peptide components ranging from 1275.9 Da–8615.5 Da [30]. Nearly 20 of these peptides are found in six lethal fractions of the anemone that induced crab paralysis, including spastic and tetanic reactions, with different degrees of intensity within seconds to several minutes. Despite this complexity, only two Kunitz-type protease inhibitors ShPI-I and ShPI-II [7,31] and four toxic polypeptides are reported in *S. helianthus*: two pore-forming cytolysins, sticholysins I and II [32]; and two ion channel blockers, Sh1 and ShK [17,20]. The cytolytic effects of the two pore-forming proteins include red blood cell hemolysis, platelet aggregation and lysis and cytotoxic and cytostatic effects on fibroblasts [33]. Lethality in mammals has been ascribed to severe vasospasm of coronary vessels, cardiac arrhythmia and inotropic effects. The toxin ShI, which specifically binds Na_v_ channels, is selectively toxic to crustaceans [20,30].

The dual effect toward protease and the ion channel detected here for ShPI-1 and reported in another five proteins from sea anemones (Table 3) has been assumed as an economic manner to save energy and an advantage for these organisms from both an offensive and defensive point of view [16].

According to [14], these molecules are survivors of a remote past in which evolution gave rise to more than one function in the same scaffold. Thus, the number of proteins acting on ion channels is rationalized, and the efficiency is increased, since the same polypeptide ensures: (i) the paralysis of the prey, though a toxic activity; and (ii) the protection against degradation of peptidic toxins injected into the prey, through the inhibition activity against proteases. Since ShPI-1 is able to inhibit a broad spectrum of serine proteases, including plasmin and kallikrein, it could ensure not only the protection of *S. helianthus* toxins, but also an additional effect on the prey’s enzymes.

### 2.3. Sequence and Structural Comparison of ShPI-1 and Other Kv Channel Toxins

**Figure 4:**
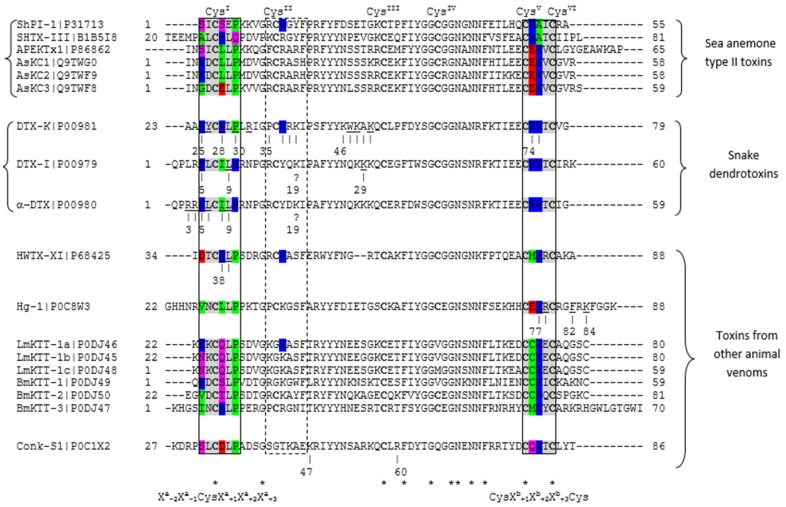
Sequence alignment of Kv-channel blocking toxins. Residues reported as crucial in the activity toward Kv channels are underlined. Conserved Cys residues are in bold and (*) represent conserved residues. Positions buried to solvent or partially exposed are highlighted in gray. Residues with a negative charge are highlighted in red; positive charge (blue); polar uncharged (magenta); and hydrophobic (green). The numbering system in the corresponding UniProt entry (protein name and UniProt accession code) is used here. Continuous line boxes enclose the sequence regions around Cys^I^ and Cys^V^-Cys^VI^, which show an amino acid substitution pattern in most of these proteins. A discontinuous line box encloses key residues around Cys^II^. Additional data about toxins included in this alignment are provided in Table 3 and Table 4. A list of the ShPI-1 interatomic contacts and the accessible surface area (ASA) relative to the residue in a vacuum is provided in the Supplementary File 1.

In a first attempt to explain Kv channel inhibition activities of ShPI-1, we carried out a sequence comparison between the five BPTI-Kunitz-type proteins from sea anemones shown in Table 3 and structurally-similar Kv channel blocking toxins from the snake *Dendroaspis polylepis.* The preliminary sequence comparison showed that most key residues reported for Kv channel inhibition in DTXs are not conserved among currently-known sea anemone toxins (Figure 4).

For example, the dyad Lys5/Leu9, crucial for the interaction of α-DTX with Kv channels [12,41], is only fully conserved in the kalicludines AsKC1 and AsKC2 [14]. This observation indicated that the functional dyad found in α-DTX and DTX-I is not needed in ShPI-1 or that this function can be fulfilled by other residues, as previously suggested for similar sea anemone toxins (*i.e.*, SHTXIII and APEKTx1, [16]).

Next, we included similar toxins from other venomous animals, some of them with known residues involved in Kv channel inhibition or with 3D structures already described. Information about all toxins used in the sequence comparison is shown in Table 3. As shown in Figure 4, the functional dyads or other key residues in DTXs and similar toxins from other venomous animals are located mainly toward the *N*- and *C*-terminal regions of the proteins, in particular around Cys^I^ and Cys^V^-Cys^VI^, respectively.

**Figure 5:**
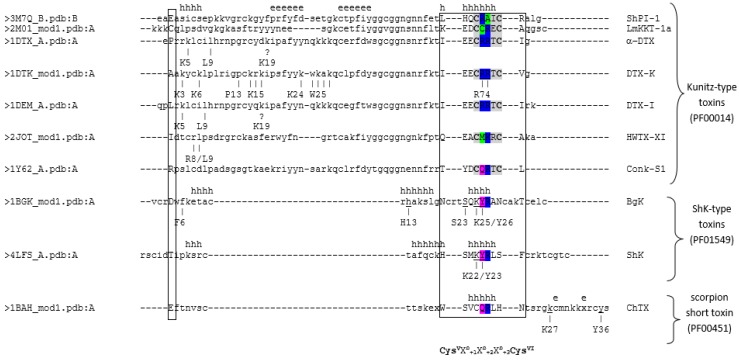
Structure-based sequence alignment of Kv-channel blocking toxins. Uppercase letters represent the equivalent position in the 3D superimposition. Continuous line boxes enclose those regions that superimpose well among toxins sharing the BPTI-Kunitz fold and those from ShK-type and scorpion-type families, also active against Kv channels. Information about their respective secondary structure elements, helix (h) and β-sheet (e), is shown over each sequence, based on ShPI-1 as representative of all BPTI-Kunitz-type proteins and the toxins ShK, BgK and ChTx. Information about toxin family, including the Pfam database (PF) code, is shown at the right. Conserved Cys residues are in bold, and positions buried to solvent or partially exposed are highlighted in gray. Residues with a negative charge are highlighted in red; positive charge (blue); polar uncharged (magenta); and hydrophobic (green).

A number of known key residues is localized outside these two regions, *i.e.*, over a β-sheet surface in the vicinity of the reactive loop needed for serine protease inhibition (Figure 4 and Figure 5). However, these data should be considered with caution. For example, Lys19 in DTX-I and α-DTX has the UniProt annotation “not important for inhibition of potassium channels”, while the equivalent Lys39 in DTX-K contains the annotation “K → A: Slight decrease in binding affinity for Kv. Important decrease in binding affinity for Kv, when associated with K48A”. In addition, the identification of key residues might be influenced by the subtype of the potassium channels used in the experiments, e.g., most of the α-DTX-susceptible Kv channels in bovine brain contain different subtypes and are composed of heterooligomeric mixtures: Kv1.2 (~80%) or Kv1.1 (~50%) [42].

Despite an apparent non-sequence conservation in key positions; we observed two subtle patterns of coordinated amino acid substitutions in different regions of Kv blocking toxins sharing a BPTI-Kunitz fold, *i.e.*, [*X*_*a*−2_*X*_*a*−1_Cys^I^*X*_*a*+1_*X*_*a*+2_*X*_*a*+3_] flanking the conserved cysteine (Cys^I^) at the *N*-terminal and [Cys^V^*X*_*b*+1_*X*_*b*+2_*X*_*b*+3_Cys^VI^] toward the *C*-terminal. Both patterns contain key residues for inhibition of potassium channels (Figure 4) and are close in space to each other, constituting a contact surface, as will be discussed later. The amino acid residues at positions *X*_*a*−1_, Cys^I^, *X*_*a*+2_, Cys^V^, *X*_*b*+3_, Cys^VI^ (shown in gray in Figure 4) are not exposed or only partially exposed in the BPTI-Kunitz fold. These residues establish a network of backbone-backbone and backbone-side chain interactions due to the conserved disulfide bridge Cys^I^-Cys^VI^ (more details in Supplementary File 1).

Therefore, we focused the analysis on fully-solvent-exposed positions (accessible surface area (ASA) > 50%) to predict amino acids important for Kv-blocking in sea anemone type II toxins. As shown in Figure 4, the fully-exposed positions *X*_*a*−2_, *X*_*a*+1_, *X*_*a*+3_, *X*_*b*+1_ and *X*_*b*+2_ contain at least one important residue in snake dendrotoxins and toxins from other venomous animals. The position *X*_*a*−2_ contains the key residues Lys5 in α-DTX and DTX-I and Lys25 in DTX-K; *X*_*a*+1_ the Ile8 in α-DTX, Lys28 in DTX-K and Arg38 in HWTX-XI; *X*_*a*+3_ the Pro30 in DTX-K; *X*_*b*+1_ the Arg74 in DTX-K; and *X*_*b*+2_ the Arg75 in DTX-K and Lys77 in Hg-1.

Replacement of Lys25, found in DTX-K, by an Asp residue (*X*_*a*−2_) in HWTX-XI, together with the absence of Trp47 and Lys48 in the latter, is proposed to explain the lower activity of the spider toxin. In DTX-K, however, the presence of Trp47 is suggested to provide a more effective hydrophobic binding surface to the Kv1.1 turret [37]. The toxins αDTX, DTX-I and DTX-K, from the dendrotoxin subfamily, possess similar IC_50_ values for Kv1.1 (Table 4). According to the alignment in Figure 4, positions *X*_*a*−2_, *X*_*b*+1_ and *X*_*b*+2_ accommodate a positive charge (Arg, Lys), but positions *X*_*a*+1_ and *X*_*a*+3_ differ between these protein isoforms or paralogous sequences. DTX-I and α-DTX contain a hydrophobic residue (Ile) in *X*_*a*+1_ and a positive charge (His) in *X*_*a*+3_. Then, a single substitution at one of these positions should be “compensated” by a second substitution to preserve the specificity in the interaction. In agreement with this criterion, DTX-K shows two coordinated substitutions in a “compensated” manner: Ile is replaced by Lys in *X*_*a*+1_, and His is replaced by Pro in *X*_*a*+3_. In this way, the balance of physical-chemical properties from residues close in space is preserved. However, specific cases where substitutions are “not compensated” should indicate that the toxin modifies its interaction specificity in comparison to protein isoforms or paralogous sequences.

Other possibilities of coordinated substitutions are observed in different subfamilies (Figure 4). Toxins from other venomous animals show conserved hydrophobic residues (Pro) at *X*_*a*+3_ and a positive charge (Arg, Lys) at *X*_*b*+2_. The other three fully-exposed positions (*X*_*a*−2_, *X*_*a*+1_, *X*_*b*+1_) in this subfamily vary in a “non-compensated” manner, which indicates that protein isoforms or paralogous sequences modify their interaction specificities. Nevertheless, some of these toxins might share the same specificity (*i.e.*, LmKTT-1a and BmKTT-1 and also LmKTT-1b and LmKTT-1c) because they preserve the distribution of hydrophobic residues (green), charged (blue, red) and polar uncharged (magenta) at equivalent positions (Figure 4).

Concerning the sea anemone type II toxins subfamily, AsKC1 and AsKC2 should have the same specificity, but different from AsKC3 and the other members. In addition, the pattern of hydrophobic residues, charged and polar uncharged is somehow similar to the pattern observed in toxins from other venomous animals (Figure 4).

It is worth mentioning that the patterns of coordinated amino acid substitutions here proposed for BPTI-Kunitz-type toxins could be masked due to: (i) a few number of reported proteins with bifunctional activity; and (ii) possibly other as yet undescribed protein isoforms or paralogous sequences in these organisms having a different pattern of coordinated amino acids substitutions. Nevertheless, the two patterns here proposed are in agreement with the widely-accepted hypothesis stating that functional residues in a protein family sharing the same fold and related functions tend to be conserved through evolution at equivalent positions [43].

On the other hand, we investigated the spatial disposition of key residues (e.g., reported dyads) among toxins having a BPTI-Kunitz fold or not, through structural superposition of these proteins using the PDBeFold method [44] (Figure 5). We observed that the *C*-terminal α-helix in proteins sharing the BPTI-Kunitz fold is the unique secondary structure element well superposed with similar helical structures found in toxins having a different fold, but displaying also activity toward Kv channels. Moreover, the proposed pattern Cys^V^*X*_*b*+1_*X*_*b*+2_*X*_*b*+3_Cys^VI^ is positioned in the *C*-terminal α-helix. Note as well that the structurally-superposed α-helices also contain the reported dyads Lys25-Tyr26 in BgK and Lys22-Tyr23 in ShK, both ShK-type toxins sharing an all-alpha fold (Figure 5).

**Figure 6:**
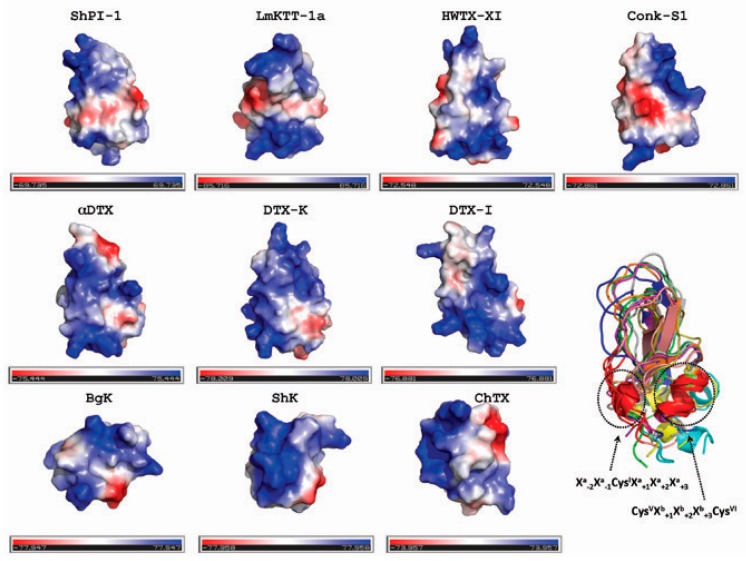
Surface electrostatic potential representation of toxins. The surface is colored according to the electrostatic potential: negative regions (in red), positive regions (in blue) and neutral regions (in gray). The orientation of the surface electrostatic potentials is the same as that in the ribbon representation at the bottom right. The list of PDB codes for toxins is as follows: ShPI-1 (3M7Q); LmKTT-1a (2M01); HWTX-XI (2JOT); Conk-S1 (1Y62); α-DTX (1DTX); DTX-K (1DTK); DTX-I (1DEM); BgK (1BGK); ShK (4LFS); and ChTX (1BAH). We also provided a color intensity scale to better represent the electrostatic potential. BPTI-Kunitz-type toxins are displayed in the first two lines. These figures were prepared with the PyMOL Molecular Graphics System, Schrödinger, LLC.

A combined analysis of the [Cys^V^*X*_*b*+1_*X*_*b*+2_*X*_*b*+3_Cys^VI^] pattern with the structure-based sequence alignment of Kv-channel blocking toxins (Figure 5) allows us to propose that the Kv channel inhibition activity here detected in ShPI-1 involves residues around positions *X*_*b*+1_-*X*_*b*+2_ in the *C*-terminal α-helix. We are aware that other residues inside or nearby the [*X*_*a*−2_*X*_*a*−1_Cys^I^*X*_*a*+1_*X*_*a*+2_*X*_*a*+3_] pattern could contribute to the interaction with the Kv channels. At the same time, this pattern creates a differential distribution of polar and non-polar groups on a common interacting surface between protein isoforms or paralogous sequences and Kv channels (Figure 6).

Previous investigations of scorpion toxins indicate that the positive ammonium group of the lysine residue in the dyad may mimic K^+^ ions entering the pore, occluding the ion pathway. A similar role is associated with the functional lysine of the toxins from sea anemones [45]. It is also proposed that the interaction with the channel occurs by the helix side of the toxin and that the β-sheet charged surface is important for a second step following toxin binding [46]. For a recent review about computational studies of venom peptides targeting Kv channels, see Chen and Chung [47]. Altogether, these results hint at the functional importance of positively-charged residues in the protein surface. In Figure 6, we present the surface electrostatic potential of the Kv channel-blocking toxins here analyzed. As seen, the distribution of positively-charged residues differs between toxins. However, a planar negatively-charged surface at the bottom is a common feature of the toxins having the BPTI-Kunitz fold (first two lines in Figure 6). Both the *N*- and *C*-terminal helices contribute to this negatively-charged surface. Interestingly, only ShPI-1, LmKKT-1a, HWTX-XI and DTX-K have a remarkable positive charge in the reactive loop that is needed by Kunitz-type proteins for the inhibition of serine proteases.

It should be noted, however, that besides the functional dyad, other toxin determinants are required for a high affinity interaction between a toxin and its target [48]. Known examples of toxins lacking a dyad, but still capable of blocking the Kv channel (or the other way around, toxins with a dyad, but incapable of blocking) strongly suggest that the functional dyad on its own cannot represent the minimal pharmacophore or prerequisite for Kv1 binding [49]. Thus, further structure-function studies, such as site-directed mutagenesis experiments, may help to determine which amino acids are the key residues for the Kv channel-inhibiting activity of ShPI-1.

## 3. Materials and Methods

### 3.1. Protein Production and Purification

The inhibitor *r*ShPI-1A was expressed in a 1.5-L working volume fermenter (B.E. Marubishi, Tokio, Japan) using the conditions previously established [8]. Secretion of the protein to the medium was evaluated by SDS-PAGE [50] and the determination of inhibitory activity against bovine pancreatic trypsin (EC 3.4.21.4) using the substrate *N*-benzoyl-arginine-*p*-nitroanilide (BAPNA) [51]. Protein purification was performed in a Streamline™ (GE Healthcare, Uppsala, Sweden) Direct HST-1 cation-exchange column according to [8]. The elution profile was monitored at 280 nm, and the inhibitory activity against pancreatic trypsin was tested as described [51]. The protein concentration was estimated by measuring the absorbance at 280 nm using the extinction coefficient (E^280^ 1% = 5.2) reported for natural ShPI-1 [7].

### 3.2. Protease Inhibition Studies

#### 3.2.1. Inhibitory Specificity toward Serine Proteases

The specificity of *r*ShPI-1A was evaluated against the following serine proteases: chymotrypsin from bovine pancreas (EC 3.4.21.1); elastase (EC 3.24.21.36) and tissue kallikrein (3.4.21.35), both from porcine pancreas; human neutrophil elastase (EC 3.4.21.37), plasmin (EC 3.24.21.7), thrombin (EC 3.24.21.5), coagulation factor Xa (EC 3.24.21.6); and *Bacillus licheniformis* subtilisin A (EC 3.4.21.62) (Table 4). All assays (*n* = 3) were performed in an Ultrospect 4000 spectrophotometer (Pharmacia Biotech, Uppsala, Sweden), under initial velocity conditions. The substrate hydrolysis was followed at 25 °C by recording the absorbance at 405 nm for 3 min with 15-s intervals.

The inhibitory activities were determined after incubating the inhibitor with the enzymes for 10–30 min at 25 °C and determining the residual enzymatic activities. One unit of inhibitory activity was defined as the amount of protein needed to inhibit one unit of enzymatic activity, which was defined as the amount of enzyme able to hydrolyze 1 µmol of substrate per min under the specified conditions. The time needed to reach inhibition equilibrium was previously established for each assay by preincubating *r*ShPI-1A with the enzymes for 1, 5, 10 or 20 min before substrate addition. Additionally, the effect of substrate concentration was determined using concentrations equivalent to 0.5 *K*_M_, 1 *K*_M_ and 2 *K*_M_.

#### 3.2.2. Determination of the Equilibrium Dissociation Constants (*Ki)*

The active concentration of trypsin was measured with a standard solution of p-nitrophenyl-*p*′-guanidinium benzoate (9.4 µM) [59]. The inhibitor active concentration was evaluated by titration with trypsin under conditions of [*E*_0_]/*Ki* ≥ 100 and assuming an equimolar enzyme:inhibitor complex. The active inhibitor concentration was assessed at the equivalence point (where [*E_t_*] = [*I_t_*]) in a plot of residual enzymatic activity *versus* the inhibitor volume. Equilibrium dissociation constants were determined by measuring the residual enzymatic activities (*v_i_*) after pre-incubation of enzymes with increasing concentrations of inhibitor under conditions of [*E*_0_]/*Ki* = 10. Apparent *Ki* values *(Ki*_app_) were obtained by adjusting the experimental points to the equation described for tight binding inhibitors [21], using non-linear fitting with GraFit v.3.01 (Erithacus Software Ltd, Horley, UK, 1997). Real *Ki* values were calculated using the equation *Ki* = *Ki*_app_/([*S*_0_]/(*K_M_*) + 1), considering the substrates concentration [*S*_0_] and their reported *K_M_* values (Table 4).

### 3.3. Voltage-Gated Ion Channel Inhibition Experiments

#### 3.3.1. Expression of Voltage-Gated Ion Channels in *Xenopus laevis* Oocytes

For the expression of the voltage-gated potassium channels (rKv1.1, rKv1.2, hKv1.3, rKv1.4, rKv1.5, rKv1.6, *Shaker* IR, hKv2.1) in *Xenopus* oocytes, the linearized plasmids were transcribed using the Ambion™ T7 or SP6 mMESSAGE-mMACHINE transcription kit (Thermofisher, Houston, TX, USA). The harvesting of Stage V–VI oocytes from an anaesthetized female *Xenopus laevis* frog was carried out as previously described [16]. Oocytes were injected with 50 nL of cRNA at a concentration of 1 ng/nL using a micro-injector (Drummond Scientific, Broomall, PA, USA). The oocytes were incubated in a solution containing: 96 mM NaCl; 2 mM KCl; 1.8 mM CaCl_2_; 2 mM MgCl_2_ and 5 mM HEPES (pH 7.4), supplemented with 50 mg/L gentamycin sulfate.

#### 3.3.2. Electrophysiological Recordings

Two-electrode voltage-clamp recordings were performed at room temperature (18–22 °C) using a Gene Clamp 500 amplifier (Molecular Devices, Sunnyvale, CA, USA) controlled by a PClamp data acquisition system (version 10, Axon Instruments, Molecular Devices, Sunnyvale, CA, USA, 2011). Whole cell currents from oocytes were recorded 1–4 days after injection. The bath solution composition was 96 mM NaCl, 2 mM KCl, 1.8 mM CaCl_2_, 2 mM MgCl_2_ and 5 mM HEPES (pH 7.4). Voltage and current electrodes were filled with 3 M KCl. Resistances of both electrodes were kept between 0.7 and 1.5 MΩ. The elicited potassium currents were filtered at 0.5 kHz and sampled at 2 kHz using a four-pole low-pass Bessel filter (Molecular Devices, Sunnyvale, CA, USA). Leak subtraction was performed using a −P/4 protocol. K_V_1.1–K_V_1.6 and *Shaker* currents were evoked by 250-ms depolarizations to 0 mV followed by a 250-ms pulse to −50 mV, from a holding potential of −90 mV. For K_V_2.1, currents were elicited by 500-ms pulses to +20 mV from a holding potential of −90 mV. In order to investigate the current–voltage relationship, current traces were evoked by 10-mV depolarization steps from a holding potential of −90 mV. To assess the concentration dependency of the toxin-induced inhibitory effects, a concentration-response curve was constructed, in which the percentage of current inhibition was plotted as a function of toxin concentration. Data were fitted to the Hill equation: *y* = 100/[1 + (IC_50_/[toxin])*h*], were *y* is the amplitude of the toxin-induced effect, IC_50_ is the toxin concentration at half-maximal efficacy, [toxin] is the toxin concentration and *h* is the Hill coefficient. A comparison of two sample means was made using a paired Student’s *t* test (*p* < 0.05). All data represent at least 3 independent experiments (*n* ≥ 3) and are presented as the mean ± standard error.

### 3.4. Computational Analyses

Sequences and three-dimensional (3D) structures of ShPI-1 and other toxins here analyzed were retrieved from the UniProt/Swiss-Prot and the Protein Data Bank (PDB) databases respectively. ClustalOmega [60] was used for multiple sequence alignment. Structural comparisons were performed using PDBeFold [44]. The interatomic contacts and accessible surface area (ASA) relative to the residue in a vacuum were calculated using WHAT IF [61].

## Figures and Tables

**Table 1:** Specificity and *Ki* values of *r*ShPI-1A against serine proteases.

Enzyme	*I*_0_/*E*_0_	Residual Activity (%)	*Ki* Values (nM)
Bovine pancreatic trypsin	0.2	63	(2.7 ± 0.3)
Human plasmin	0.3	64	(4.4 ± 0.2)
Porcine pancreatic kallikrein	0.6	63	(14.6 ± 2.00)
Human factor *Xa*	^a^	100	N.i ^b^
Human α-thrombin	^a^	100	N.i ^b^
Porcine pancreatic chymotrypsin	0.9	52	(14.8 ± 1.5)
Human neutrophil elastase	0.6	66	(23.5 ± 2.0)
Porcine pancreatic elastase	1–170	100	N.i ^b^
*B. licheniformis* subtilisin	1–240	100	N.i ^b^

^a^ Enzyme amount was provided in enzymatic units (see Materials and Methods), and therefore, molar concentrations were unavailable. ^b^ N.i means that no inhibition was detected, even increasing the ratios of inhibitor *vs*. enzyme molar concentration (*I*_0_/*E*_0_) and the incubation times up to 30 min.

**Table 2:** Sea anemone proteins with bifunctional activity, toward serine proteases and Kv channels.

Organism	Inhibitor	UniProt	Antiprotease *Ki* (nM)	Kv Inhibition ^a^ (IC_50_)	Reference
*Stichodactyla helianthus*	ShPI-1	P31713	See Table 1	117 ± 15 nM (Kv1.1)	this study
				9 ± 2 nM (Kv1.2) + (Kv 1.6)	
*Anemonia sulcata*	AsKC1	Q9TWG0	Tr: <30	2.8 µM/Kv1.2	[14]
	AsKC2	Q9TWF9	Tr: <30	1.1 µM/Kv1.2	
	AsKC3	Q9TWF8	Tr: <30	1.3 µM/Kv1.2	
*Anthopleura elegantissima*	APEKTx1	P86862	Tr: 120	0.9 nM/Kv1.1	[16]
*Stichodactyla haddoni*	SHTX-3	B1B5l8	Tr: 203 IU/mg	0.65 mM ^b^	[15]
				(270 nM) ^c^	

Tr: Trypsin. Empty spaces mean activities not tested or referred to. ^a^ Specificity towards different K^+^ channel subtypes is included if available; ^b^ crab-paralyzing activity determined using synaptosomal binding assay; ^c^ competitive binding to I^125^-αDTX.

**Table 3:** BPTI-Kunitz-type Kv channel toxins from non-anemones animal venoms.

Taxon	Toxin	UniProt	PDB	Target (IC_50_, nM)	Key Residues *	Reference
snakes	α-DTX	P00980	1DTX	Kv1.1 (1.1–150)	R3, R4, K5, L6, I8, L9	[13]
				Kv1.2 (0.4)		
				Kv 1.6 (9.0)		
	DTX-K	P00981	1DTK	Kv1.1 (2.5) (0.03)	K25, Y26, K28, P30, R32, P35, K37-K39, K46-K50, R74, R75	[34,35]
	DTX-I	P00979	1DEM 1DEN	Kv1.1 (3.1)	K5, L9, K29	[13]
				Kv1.2 (0.13)		
				Kv1.6; Kv1.3; Kv1.5 (weaker)		
scorpion	Hg1	P0C8WT	-	Kv1.3 (6.2)	*C*-terminal (K77, R78, F82, K84)	[36]
	LmKTT-1a	P0DJ46	2M01	Kv1.3 (>1000)	-	[36]
	LmKTT-1b	P0DJ45	-	Kv1.3 (>1000)	-	[36]
	LmKTT-1c	P0DJ48	-	Kv1.3 (>1000)	-	[36]
	BmKTT-1	P0DJ49	-	Kv1.3 (129.7)	-	[36]
	BmKTT-2	P0DJ50	-	Kv1.3 (371.3)	-	[36]
	BmKTT-3	P9DJ47	-	Kv1.3 (>1000)	-	[36]
spider	HWTX-XI	P68425	2JOT	Kv1.1 (2.6 mM)	R38-L39	[37]
				Kv1.2; 1.3 (weaker)		
*Conus* mollusk	Conk-S1	P0C1X2	2CA7	Kv1 (60)	No dyad needed; K47, R60	[38,39,40]
			1Y62	Kv1.7 (439)		

* Based on site-directed mutagenesis studies and using the numbering system at UniProt. Residues referred in the literature as “functional dyads” are underlined. The symbol (-) means unavailable data.

**Table 4:** Experimental conditions for serine protease inhibition assays.

Enzyme	[*E*_0_] ^1^ (M or U)^2^	Substrate (Reference)	[*S*_0_] (mM) ^2^	Buffer
trypsin	2.2 10^−7^	Bz-Arg-*p*Na [51]	1.0	20 mM Tris-HCl; 150 mM NaCl; 20 mM CaCl_2_; pH 8.0
kallikrein	5.4 10^−7^	HD-Val-Leu-Arg-pNA [52]	0.5	50 mM Tris-HCl; 16 mM NaCl; pH 7.8
plasmin	2.4 10^−8^	HD-Val-Leu-Lys-pNA [53]	0.4	50 mM Tris-HCl; 110 mM NaCl; pH 7.4
factor Xa	0.1 U	Bz-Ile-Glu-Gly-Arg-pNA [54]	0.4	50 mM Tris-HCl; 130 mM NaCl; 20 mM CaCl_2_; pH 8.3
thrombin	1.0 U	HD-Phe-Pip-Arg-pNA [55]	0.4	50 mM Tris-HCl; 130 mM NaCl; 20 mM CaCl_2_; pH 8.3
chymotryp.	2.8 10^−8^	Suc-Ala-Ala-Pro-Phe-*p*NA [56]	1.0	50 mM Tris-HCl; pH 8.0
neutrophil elastase ^2^	1.0 10^−7^	MeOSuc-Ala-Ala-Pro-Val-*p*NA [57]	0.14	20 mM Tris-HCl; 500 mM NaCl; pH 8
pancreatic elastase	3.3 10^−8^	Suc-Ala-Ala-Ala-*p*NA [57]	0.7	30 mM Sodium Phosphate; 50 mM NaCl; pH 7.0
subtilisin A	2.3 10^−9^	Suc-Ala-Ala-Pro-Phe-*p*NA [58]	0.12	100 mM Tris-HCl; 0.1% Triton X-100 ; pH 8.6

^1^ Abbreviations used: [*E*_0_], enzyme concentration; U, enzyme activity units; [*S*_0_], substrate concentration, around 1 *K*_M_ in all enzymes; *K*_M_, Michaelis constant. ^2^ Concentrations in the assays. One unit of enzymatic activity was defined as the amount of enzyme able to hydrolyze 1 µmol of substrate per min under the specified conditions.

## Supplementary Materials

The following is available online at www.mdpi.com/2072-6651/8/4/110/s1: the Supplementary File 1.

## Abbreviations

The following abbreviations are used in this manuscript:

3D: three-dimensional
ASA: accessible surface area
BAPNA: *N*-benzoyl-arginine-*p*-nitroanilide or Bz-Arg-pNA
BPTI: bovine pancreatic trypsin inhibitor
DTX: dendrotoxin
*Ki*: equilibrium dissociation constant
Kv: voltage-gated potassium channel
SDS-PAGE: sodium dodecyl sulfate-polyacrylamide gel electrophoresis
ShPI-1: *Stichodactyla helianthus* protease inhibitor 1
